# Techno-economic and Sustainability Assessment of Integrated Annatto Seed Biorefinery for Natural Pigments, Starch, and Nutraceuticals

**DOI:** 10.1021/acsenvironau.5c00207

**Published:** 2026-02-18

**Authors:** Giovani Leone Zabot, Eric Keven Silva

**Affiliations:** † Laboratory of Agroindustrial Processes Engineering (LAPE), 28118 Federal University of Santa Maria (UFSM), 3013 Taufik Germano Rd, Universitário II DC, Cachoeira do Sul - RS 96503-205, Brazil; ‡ Faculdade de Engenharia de Alimentos (FEA), 28132 Universidade Estadual de Campinas (UNICAMP), Rua Monteiro Lobato, 80, Campinas, São Paulo CEP 13083-862, Brazil

**Keywords:** Economic analysis, Supercritical CO_2_ extraction, Ultrasound-assisted extraction, Starch recovery, Sustainability metrics

## Abstract

Annatto (*Bixa orellana* L.) seeds, traditionally exploited as a natural pigment source, remain underused despite their richness in bioactive oil and functional carbohydrates. We propose an integrated biorefinery that couples supercritical CO_2_ oil extraction, ultrasound- and low-pressure solvent-assisted bixin recovery, aqueous starch isolation, and oil encapsulation into nutraceutical powders. Experimental data were scaled with SuperPro Designer to quantify techno-economic performance, and sustainability was assessed using a 12-principle green-extraction metric (Path2Green) to benchmark process choices across environmental, economic, and scalability dimensions. The integrated process achieved a gross margin of 21.8%, ROI 27.9%, NPV USD 5.8 million (7% rate), and a 3–4 year payback time; sensitivity analysis identified feedstock cost and product price as the dominant profitability drivers, while labor and utilities had comparatively minor effects. Sustainability results highlighted the superiority of supercritical CO_2_ over Soxhlet for oil (solvent safety/recyclability), energy trade-offs between ultrasound and low-pressure solvent extraction for bixin, and the valorization potential of aqueous starch recovery. Collectively, the findings position annatto as a model biomass for zero-waste, circular biorefineries delivering natural pigments, functional carbohydrates, and nutraceutical ingredients through processes that are both economically viable and metrics-driven in sustainability.

## Graphical abstract

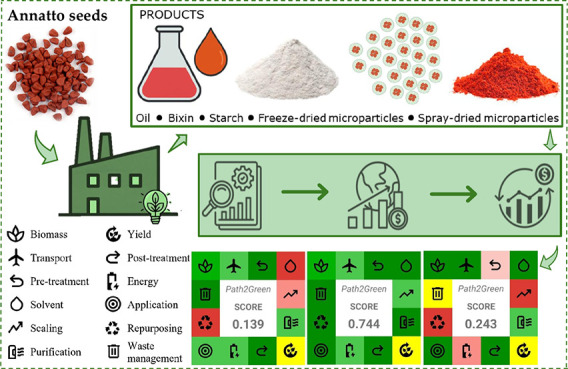

## 1 Introduction

The growing demand for natural colorants, functional ingredients, and sustainable bioprocesses has intensified the search for renewable feedstocks that can be valorized within integrated biorefineries. Conventional single-product extraction approaches often overlook valuable coproducts, resulting in inefficiencies and underexploited biomass potential. In this context, tropical seeds and byproducts represent attractive raw materials due to their phytochemical diversity, functional carbohydrates, and bioactive compounds.^1^ However, to establish them as competitive alternatives in the global market, it is essential to couple innovative extraction technologies with rigorous assessments of economic feasibility and sustainability metrics.

Annatto (*Bixa orellana* L.), commonly known as achiote or roucou, is a perennial shrub native to Central and South America that has been cultivated for centuries as one of the world’s most important natural sources of carotenoids. Traditionally recognized for its reddish-orange pigment, annatto seeds are the primary source of bixin, a natural apocarotenoid widely used as a food colorant (E160b) in dairy, bakery products, vegetable oils, and beverages. Beyond its use as a dye, bixin and norbixin have attracted attention for their antioxidant, anti-inflammatory, and anticancer properties, reinforcing their role as bioactive ingredients in nutraceuticals and pharmaceuticals.^2,3^

In addition to pigments, annatto seeds contain other high-value compounds, notably an oil fraction rich in geranylgeraniol, recognized for its pharmacological activities, including anti-inflammatory, hypoglycemic, and anticancer effects, and tocotrienols, especially δ- and γ-tocotrienol, known for their antioxidant and cholesterol-lowering properties.^4^ Furthermore, the depigmented seed biomass contains starch with promising functional and technological properties, which remains largely underexplored.^5,6^ Thus, annatto represents not only a traditional dye source but also a multipurpose biomass for developing integrated biorefinery processes that valorize pigments, bioactive-rich oil, and carbohydrates simultaneously.

Traditionally, industrial processing of annatto seeds has relied on solvent-intensive methods such as Soxhlet extraction for oil recovery or alkaline extraction for pigments. While effective, these approaches raise concerns regarding solvent residues, energy consumption, and environmental impacts.^7^ At the same time, they focus primarily on single-product recovery, leaving valuable fractions underutilized. Recent advances in green technologies, such as supercritical CO_2_ extraction (SFE), ultrasound-assisted extraction (UAE), and low-pressure solvent extraction (LPSE), provide alternatives that align with clean-label demands while enabling higher efficiency and reduced environmental footprint.^3,8,9^ Moreover, structuring annatto seed oil into nutraceutical emulsions, followed by spray- or freeze-drying, extends its functionality into stabilized dietary supplements, further diversifying potential applications.^10,11^ In this regard, Figure 1 was presented to depict a biorefinery proposed herein.

**Figure 1:**
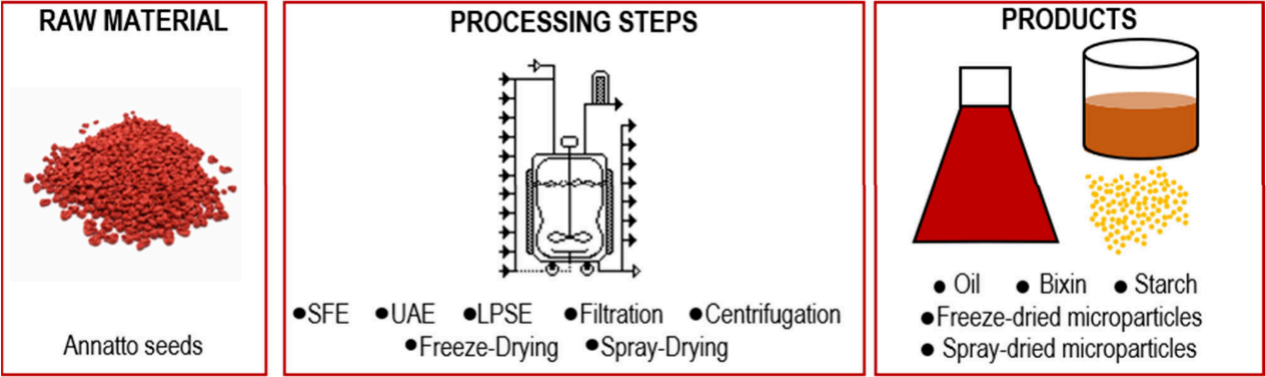
Illustration of a biorefinery scheme for processing annatto seeds using green technologies.

Despite the broad phytochemical diversity and demonstrated bioactivity of *B. orellana*, few studies have assessed its valorization within the framework of integrated biorefineries. In particular, systematic evaluations of economic feasibility and sustainability metrics are lacking, even though such analyses are critical to determine whether annatto-based processes can be competitive and environmentally responsible at scale.^12^ Considering the growing global demand for natural colorants, functional ingredients, and plant-based nutraceuticals, developing a comprehensive valorization pathway for annatto is both timely and strategically important.

Therefore, the present study proposes and evaluates an integrated annatto seed biorefinery comprising (*i*) supercritical CO_2_ extraction of seed oil, (*ii*) ultrasound- and low-pressure solvent-assisted extraction of bixin, (*iii*) aqueous starch recovery, and (*iv*) encapsulation of oil into nutraceutical powders. The integrated process was subjected to techno-economic evaluation using SuperPro Designer and to a sustainability assessment through the Path2Green framework. Together, these analyses provide a holistic perspective of the profitability, robustness, and environmental performance of annatto valorization, positioning this tropical crop as a model biomass for sustainable biorefining. We hypothesize that integrating supercritical CO_2_ extraction, ultrasound- and solvent-assisted pigment recovery, and aqueous starch isolation into a single annatto seed biorefinery will not only improve profitability but also align with sustainability metrics by maximizing biomass utilization, minimizing waste, and ensuring environmental responsibility.

## 2 Materials and Methods

### 2.1 Process Integration

Annatto (*Bixa orellana* L.) seeds were acquired from a local market at Campinas (Brazil). They were used for obtaining different products under laboratorial conditions using process integration: oil by supercritical fluid extraction (SFE) with CO_2_,^7,8^ bixin by ultrasound-assisted extraction with ethanol, starch by mechanical stirring and rotary drying,^5^ remaining bixin from the fine particles after milling by low pressure solvent extraction,^9^ and an emulsified powder containing oil by ultrasound-assisted emulsification followed by freeze-drying^10^ and spray-drying.^13^ Based on the experimental findings obtained after integrating the processes, this work assessed the main economic parameters, which are described in the following sections.

### 2.2 Process Description

The process pathways are presented in Figure 2. First, the annatto seeds are loaded into a high-pressure vessel and the superior lid is closed. Thereafter, CO_2_ is pressurized and the vessel is heated to achieve the desired conditions of the system. A semicontinuous extraction occurs, while the oil + CO_2_ is directed to the separation vessel. At the end of the extraction, defatted seeds are unloaded from the vessel and used in two paths, while the oil is used in another path for microparticles production.

**Figure 2:**
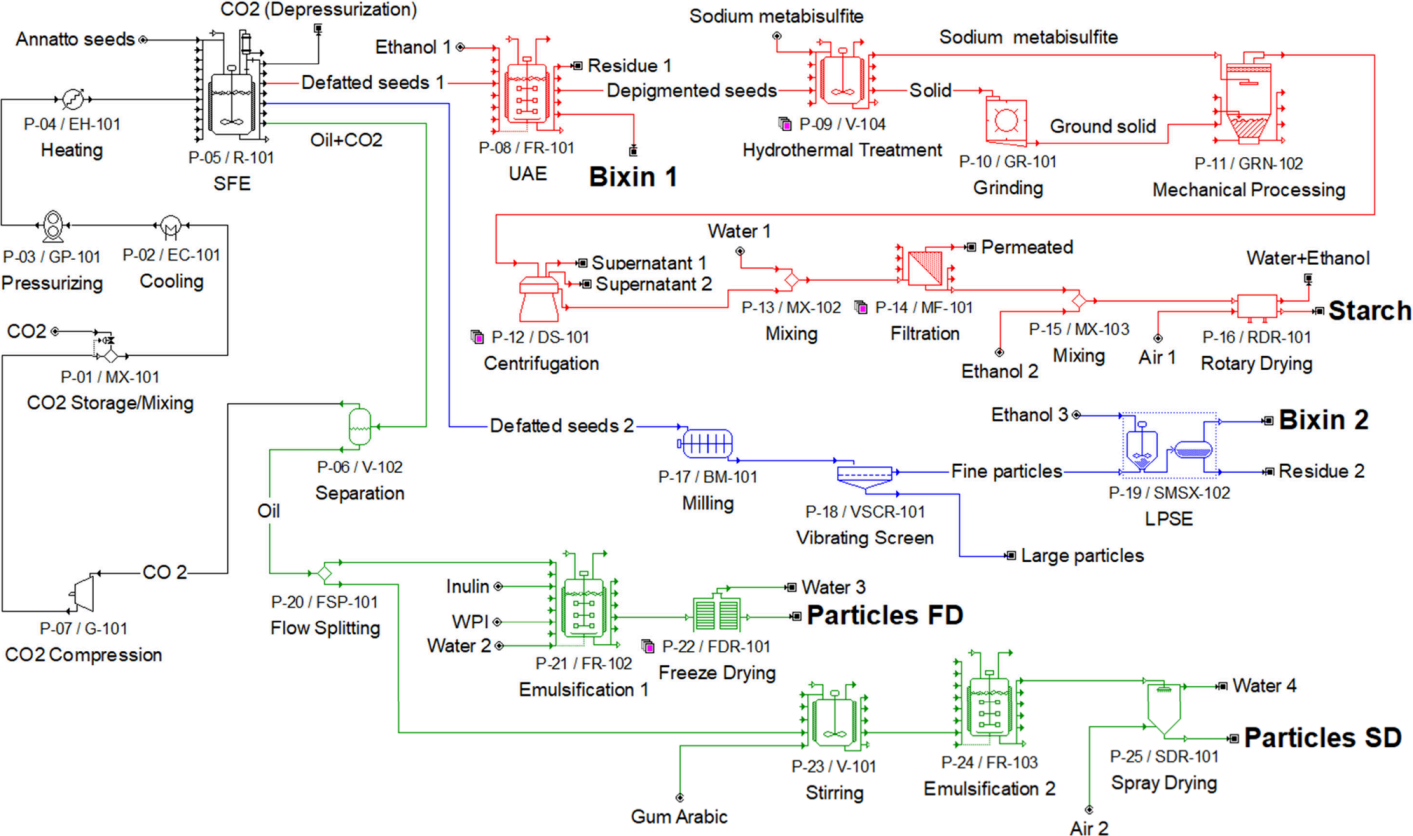
Flowsheet of the integrated processes designed in the SuperPro Designer for economic analysis of annatto seeds processing.

The first path (red lines) is the ultrasound-assisted extraction (UAE), in which the defatted seeds are mixed with ethanol and submitted to ultrasound waves. The bixin (**Bixin 1**) is extracted and the remaining solid material is the depigmented seeds. Therefore, these seeds are mixed with sodium metabisulfite. The solid material under suspension is ground and sieved. The retained material is mixed again with sodium metabisulfite (reused) and submitted to mechanical processing. The material is centrifuged and the supernatant is discarded. The precipitate is recovered with distilled water and mixed with ethanol. The starch is filtered and dried to form a powder (**starch**).

In the second path (blue lines), the defatted seeds are milled and the material is classified according to particle size using a sieve. The fraction with particle diameter lower than 48 mesh (dp ≤ 300 μm; fine particles) is submitted to low-pressure solvent extraction (LPSE) with ethanol. After this LPSE, the bixin (**Bixin 2**) is filtered through a filtration system using filter paper and the solvent is evaporated. Otherwise, the fraction with particle diameter larger than 48 mesh (dp >300 μm; coarse particles) is separated, which can be submitted to a chemical modification process using pressurized hot water to obtain annatto flour.

The third path (green lines) is the emulsification and microencapsulation of annatto seed oil by ultrasound. Inulin, whey protein isolate (WPI), and gum Arabic are the polymers used for this process. Thereafter, the emulsions formed with inulin and WPI are frozen in aluminum plates and subjected to freeze-drying. The emulsions formed with gum Arabic are dried in a spray dryer with an injection spray system with air pressure and temperature of 0.4 MPa and 170 °C. In the sequence, the dried emulsions are converted into fine powders (**particles FD** (freeze-drying) and **particles SD** (spray-drying)) through maceration.

### 2.3 Operational Parameters

The operational parameters of the processes are presented in Table 1. The selected scale-up criterion consisted of using scalable parameters, such as temperature, pressure, time, solvent to feed mass ratio (S/F), energy density, and bed density, among others. The processes developed on larger scales were assumed to have the same performance as those reached in laboratory scale. Therefore, the same yields and scalable results were used. The plant design capacity was assumed to process one tonne of annatto seed per batch, reaching 990 tons of seeds processed per year (industrial capacity).

**Table 1:** Process Parameters for Annatto Seed Processing and Results

Parameters									Results				
Process	Operation	Solvent/reagent	Temperature (°C)	Pressure (MPa)	S/F (g solvent (g material)^‑1^)	Time (min)	Bed apparent density (kg m^‑3^)	Energy density (kJ cm^‑3^)	Extract (g (kg annatto)^‑1^)	Bixin (g (g annatto)^‑1^)	Starch (g (g depigmented seeds)^‑1^)	Microparticles (g (g emulsion)^‑1^)	Reference
SFE	P-05/R-101	CO_2_	40	20	2.1	60	700	-	1.8^a^	-	-	-	^7^,^8^
UAE	P-08/FR-101	Ethanol	-	0.1	5	5	-	-	-	0.124	-	-	^3^
HT	P-09/V-104	Sodium metabisulfite 500 ppm	25	0.1	10	960	-	-	-	-	-	-	^5^
Mechanical processing	P-11/GRN-102	Sodium metabisulfite 500 ppm	25	0.1	10	60	-	-	-	-	-	-	^5^
Drying	P-16/RDR-101	-	45	0.01	-	120	-	-	-	-	0.660	-	^5^
LPSE	P-19/SMSX-102	Ethanol	40	0.1	20	45	-	-	-	0.100	-	-	^9^
Emulsification 1	P-21/FR-102	Inulin + WPI	41.6	0.1	4	5	-	7.7	-	-	-	-	^10^
Freeze-drying	P-22/FDR-101	-	–40	0.01	-	1440	-	-	-	-	-	0.250	^10^
Emulsification 2	P-24/FR-103	Gum Arabic	44.5	0.1	5	5	-	7.7	-	-	-	-	^13^
Spray drying	P-25/SDR-101	-	170	0.4	-	480	-	-	-	-	-	0.180	^13^

a Partial composition: Delta-tocotrienols (12.3 ± 0.6 g (100 g extract)^−1^)^7^ and geranylgeraniol (25.0 ± 0.6 g (100 g extract)^−1^);^13^ HT: Hydrothermal treatment; UAE: Ultrasound-assisted extraction; SFE: Supercritical fluid extraction; LPSE: Low-Pressure solvent extraction.

### 2.4 Assumptions and Data Collection

Fixed costs are divided into total plant direct cost, total plant indirect cost, and contractor’s fees and contingency. The direct costs comprise facility installation and instrumentation, among others, while the indirect costs include fees related to the construction and implementation of the project. The fee to purchase the facility is used as the base to estimate fixed costs and is calculated using different multipliers (Table 2), according to Heinzle, Biwer and Cooney.^14^

**Table 2:** Multipliers for Direct Cost and Total Capital Investment^a^

Costs	Group	Multipliers^b^
Total Plant Direct Cost	Installation	0.47 × PC
	Piping	0.68 × PC
	Instrumentation	0.26 × PC
	Electrical	0.11 × PC
	Insulation	0.08 × PC
	Yard Improvement	0.10 × PC
	Buildings	0.18 × PC
	Auxiliary facilities	0.55 × PC
Total Plant Indirect Cost	Engineering	0.73 × PC
	Construction	0.85 × PC
Contractor’s Fee and Contingency	Contractor’s fee	0.18 × PC
	Contingency	0.26 × PC
Working Capital	-	0.53 × PC
Startup Capital	-	0.18 × PC

a PC: Purchasing cost of all machines used in the project.

b Multipliers based on data reported by Cheng and Rosentrater.^15^

The purchasing cost of each machine used in the project on a laboratory-scale was quoted locally (Table 3). Thereafter, the power rule (eq 1) was used to estimate machine cost on larger scales for different production capacities^16^ because the cost of a specific item is a function of size, materials of construction, design pressure, and design temperature. The base costs listed in Table 3 are laboratory-referenced values used solely for scaling relative differences using the power law. They do not represent commercial sanitary systems and represent values of laboratory-scale equipment used for processing 1 kg of annatto seeds per batch.

1 $$C_{2}=C_{1}{\left(\frac{Q_{2}}{Q_{1}}\right)}^{n}$$

Where: C_1_ is the known base cost for equipment with capacity Q_1_, C_2_ is the equipment cost with capacity Q_2_, and n is a constant depending on the machine type.

**Table 3:** Base Cost for Machines and Devices Composing the Project for Processing 1 kg of Annatto Seeds Per Batch

Item (quantity)	n^a^	Laboratory unit base cost (USD)
Jacketed high-pressure extraction vessel (1)	0.82	1000.00
CO_2_ pump (1)	0.55	3910.00
Cooler (1)	0.59	800.00
Heater (1)	0.59	200.00
Separation vessel (1)	0.49	650.00
Compressor (1)	0.55	450.00
Storage tank (1)	0.49	250.00
Ultrasound device (1)	0.60	2200.00
Tank with agitation and heating for hydrothermal treatment (16)	0.54	300.00
Mill (2)	0.60	150.00
Device for mechanical processing (1)	0.60	550.00
Centrifuge (3)	0.49	490.00
Filtration system (3)	0.55	100.00
Rotary dryer (2)	0.40	1100.00
Vibrating screen (1)	0.40	110.00
Apparatus for low-pressure solvent extraction (1)	0.60	870.00
Emulsifier device (2)	0.60	850.00
Freeze-dryer (3)	0.60	3730.00
Spray-dryer (1)	0.60	4800.00

a n constant depending on each machine type.^16,17^

Material, facility maintenance, labor, and utility costs are the main sources of operating costs. Annatto seeds, CO_2_, ethanol, sodium metabisulfite, inulin, whey protein isolate (WPI), gum Arabic, and water are material costs. Electricity is the main energy used to operate facilities, while steam is used as a heat transfer agent in the process. Labor costs are divided into machine operators, extraction workers, and material workers. All these costs are presented in Table 4 as average costs from the last five years (2021–2025). Annatto seed particles (**particles FD** and **particles SD**), starch, and bixin (**bixin 1** and **bixin 2**) are products of the whole producing line. The estimated selling prices are also presented in Table 4.

**Table 4:** Operating Cost Inputs

Group	Description	Cost/price
Materials	Annatto seeds	2.78 (USD kg^–1^)
	Industrial CO_2_ ^a^	2.30 (USD kg^–1^)
	Industrial ethanol^a^	1.50 (USD L^–1^)
	Water (for direct use in the process)	4.00 (USD ton^–1^)
	Sodium metabisulfite	6.50 (USD kg^–1^)
	Inulin	13.50 (USD kg^–1^)
	Whey protein isolate (WPI)	9.60 (USD kg^–1^)
	Gum Arabic	9.20 (USD kg^–1^)
Labor	Machine operator	10.00 (USD (h worker)^−1^)
	Extraction worker	12.50 (USD (h worker)^−1^)
	Material worker	6.80 (USD (h worker)^−1^)
	Administrative worker	5.50 (USD (h worker)^−1^)
Utility	Electricity	0.25 (USD (kW h)^−1^)
	Steam	12.00 (USD MT^–1^)^b^
	Water (for cleaning)	1.00 (USD ton^–1^)
Products	Annatto seed particles	3.00 (USD (100 g)^−1^)
	Starch	2.00 (USD kg^–1^)
	Bixin	2.50 (USD (100 g)^−1^)

a Solvents reused in the process – ethanol: 49.9% (mass basis); CO_2_: 83.6% (mass basis).

b MT: metric tons.

Gross profit (GP), gross margin (GM), net profit (NP), and return on investment (ROI) were calculated directly in the SuperPro Designer 9.0 software (Intelligen Inc., Scotch Plains, NJ, USA) following eqs 2–5. The annual operating cost (USD year^–1^) and payback time (year) were also estimated. Payback time is the number of years to recover the initial investment. As soon as the investment is recovered, the project is profitable. Payback occurs at the moment when the sum of the terms of the cash flow is positive. These parameters are the most common indicators to assess the profitability of a new project.

$$\mathrm{Gross Profit}\;({\mathrm{USD}\;\mathrm{year}}^{-1})=\mathrm{Total Revenue}-(\mathrm{Total Operating cost}-\mathrm{credits})$$ 2

3 $$\mathrm{Gross Margin}\;\left(\%\right)=\frac{\mathrm{Gross Profit}}{\mathrm{Revenue}}\times 100$$

4 $$\mathrm{Net Profit}\;\left({\mathrm{USD year}}^{-1}\right)=\mathrm{Gross Profit}-\mathrm{Taxes}+\mathrm{Depreciation}$$

5 $$\mathrm{Return on investment}\;\left(\%\right)=\frac{\mathrm{Net Profit}}{\mathrm{Total Capital Investment}}\times 100$$

### 2.5 Economic Simulation

The simulation flowsheet and Gantt chart were performed using SuperPro Designer 9.0 software (Intelligen Inc., Scotch Plains, NJ, USA). Considering the process integration, a flowsheet was designed to evaluate the economic feasibility of processing annatto seeds to obtain the three main products: bixin, starch, and particles containing encapsulated oil (Figure 2). Experimental input data were used based on Table 1. The processes were designed to operate for 7920 h per year, which corresponds to 3 daily shifts for 330 days per year. The yearly remaining time was considered for cleaning and equipment maintenance.

The simulation software automatically performs detailed mass and energy balances for each unit operation, including CO_2_ compression and recycle, ethanol recovery, ultrasound power, freezing loads, freeze-drying sublimation energy, and spray-drying evaporation duties. These balances generate step-level utility demands (electricity, steam, water), which are converted into costs using the reference prices provided in Table 4. Equipment efficiencies are incorporated according to the standard values embedded in the software, ensuring consistency across all unit operations. Consequently, the cost of utilities (CUT) reported in Figure 3 reflects the aggregated demands computed from these internal balances. For the sensitivity analysis in Figure 4, variations of ± 5, ± 20, and ± 35% were applied to the referenced operating costs listed in Table 3, with all responses recalculated after the software executed the full mass- and energy-dependent procedures.

**Figure 3:**
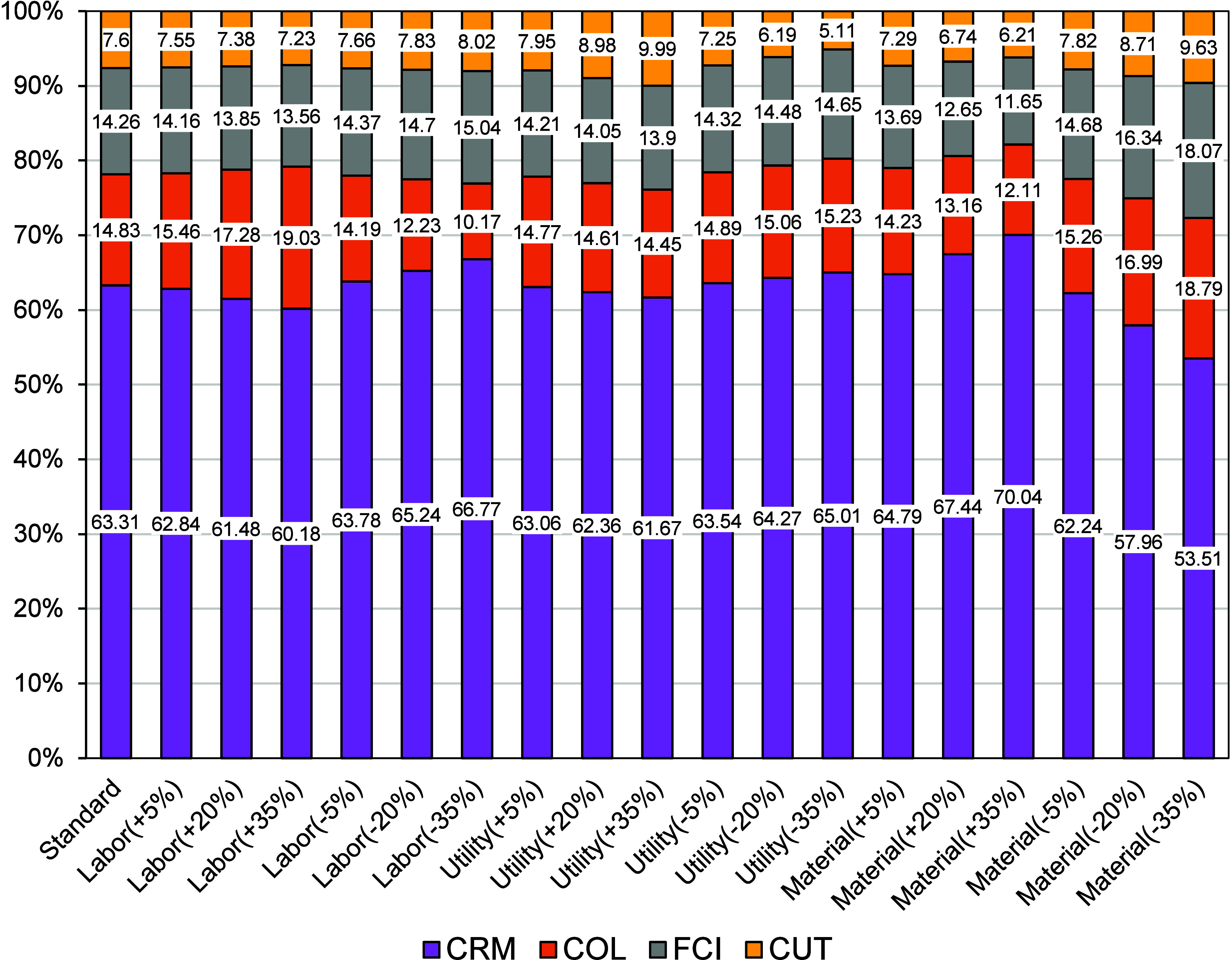
Breakdown of the operating costs for the scenarios evaluated for processing one ton of annatto seeds per batch.

**Figure 4:**
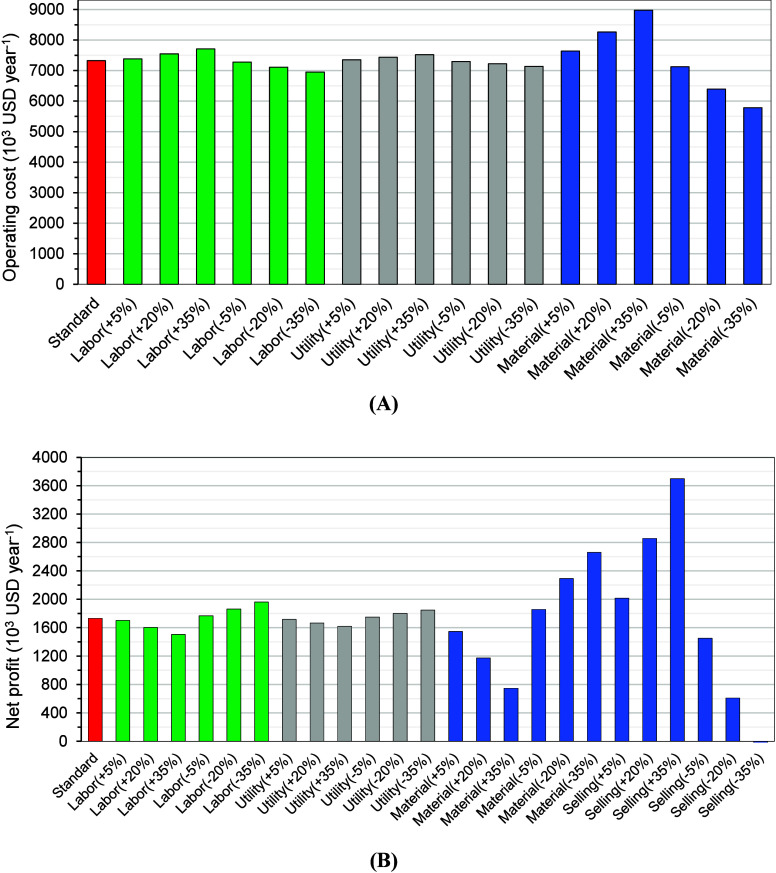
Operating cost (A) and net profit (B) for the scenarios evaluated for processing one ton of annatto seeds per batch.

### 2.6 Sensitivity Analysis

Sensitivity analysis is performed to observe the fluctuation of economic conditions and evaluate the factor that has the most significant effect on changing net profits (and the other main parameters) of a process with fluctuating economic conditions and price uncertainties. Changes in operating costs of ± 5, ± 20, and ± 35% were applied, including material, labor, and utility. Also, revenues from products were analyzed and compared with operating costs.

### 2.7 Assessment of Process Sustainability by Path2Green Metric

The sustainability of the extraction processes used for obtaining natural pigments and starch from the annatto processing chain was assessed using the Path2Green metric proposed by de Souza Mesquita, Contieri, e Silva, Bagini, Bragagnolo, Strieder, Sosa, Schaeffer, Freire, Ventura, Coutinho and Rostagno.^18^ This methodology is based on 12 green extraction principles that collectively consider environmental, social, and economic aspects of biomass valorization, including parameters such as biomass origin, transport, pretreatment, solvent choice, scalability, purification, yield, energy consumption, waste management, and possibilities for repurposing and application.

Each principle is scored on a scale from −1.0 to +1.0 according to specific criteria, and weighted according to its relevance to the three sustainability pillars, with greater emphasis placed on environmental aspects. The scores obtained for the different principles are then integrated into a single, user-friendly sustainability index, allowing the comparison of different extraction strategies.

In this study, the Path2Green assessment was applied exclusively to the extraction steps related to (*i*) annatto seed oil (extract), (*ii*) bixin (natural pigments) from annatto seeds and (*iii*) depigmented starch recovery from annatto processing residues. These processes were selected because they involve solvent- and energy-dependent operations, making them directly aligned with the scope of the metric. Other process steps, such as encapsulation of annatto seed oil, were not included in the Path2Green evaluation, since they go beyond the intended application of the metric. It is important to note that the Path2Green assessment is intentionally limited to process-level indicators within the extraction stages. As a principle-based metric, Path2Green does not account for upstream and downstream environmental impacts such as energy production, equipment manufacturing, infrastructure, or end-of-life stages. Consequently, energy-intensive technologies, including supercritical CO_2_ extraction, may exhibit additional environmental burdens beyond the system boundaries considered in this study.

By applying Path2Green, it was possible to systematically analyze the sustainability of the extraction processes, highlighting strengths and potential trade-offs in comparison with conventional benchmarks. This approach provides a transparent and reproducible basis for discussing the environmental relevance of the proposed integrated valorization chain. This work does not attempt a full cradle-to-gate life cycle assessment (LCA). Future studies should quantify greenhouse gas (GHG) emissions, cumulative energy demand, and water use.

In this work, the term “conventional benchmarks” refers to well-established extraction processes traditionally reported for annatto seed valorization. Specifically, Soxhlet extraction using hexane was adopted as the conventional benchmark for annatto seed oil recovery, due to its widespread use as an exhaustive reference method in laboratory and industrial assessments. For pigment recovery, conventional solvent-based extraction routes using ethanol or alkaline media, as commonly described in the literature, were considered as benchmarks. These reference processes were selected because they represent standard practices against which emerging green technologies are frequently compared, despite their recognized limitations regarding solvent safety, energy consumption, and scalability.

### 2.8 Scale-Up Considerations, Assumptions, and Uncertainty Treatment

Industrial performance of extraction and drying technologies frequently deviates from laboratory results due to nonideal mass and heat transfer, changes in bed hydrodynamics, solvent recovery efficiency, and energy requirements. For this reason, the techno-economic model presented here should be interpreted as a Class-4 feasibility-level techno-economic assessment, consistent with U.S. Department of Energy and National Renewable Energy Laboratory guidelines and preliminary engineering standards.

Laboratory extraction yields, solvent-to-solid ratios, and drying efficiencies were used for scaling because pilot-scale data are not yet available. Accordingly, scale-up uncertainty was incorporated conceptually by interpreting the sensitivity ranges (±5%, ± 20%, ± 35%) as realistic variance bounds for solvent consumption, yields, and utilities. These assumptions do not reflect performance guarantees but provide decision-support for the viability of an integrated annatto biorefinery. Future work should experimentally validate SFE, UAE, LPSE, ethanol recovery, and freeze/spray drying at pilot scale to replace these feasibility-level assumptions with measured industrial efficiencies.

## 3 Results and Discussion

### 3.1 Integrated Process and Product Yields

The proposed biorefinery scheme was designed to exploit annatto seeds in a sequential and integrated manner, enabling the recovery of oil, pigments, starch, and nutraceutical encapsulates within a single valorization chain. The process flowsheet (Figure 2) illustrates how high-pressure extraction, solvent-based operations, mechanical treatment, and drying steps can be strategically combined to maximize biomass utilization and minimize waste generation. Unlike conventional single-product routes, this approach distributes the raw material across multiple product streams, thereby enhancing economic resilience and aligning with zero-waste and circular economy principles.

The experimental yields obtained at laboratory scale and applied in the simulation are summarized in Table 1. Supercritical CO_2_ extraction of annatto seeds yielded 1.8 g oil per 1000 g of seeds, corresponding to an extract particularly rich in δ-tocotrienol (12.3 g (100 g)^−1^) and geranylgeraniol (18.9 g (100 g)^−1^).^7,13^ These bioactives are highly valued in the nutraceutical sector for their antioxidant, anti-inflammatory, and cholesterol-lowering properties, positioning the oil as a premium product in functional markets. Following oil recovery, the defatted seeds served as a feedstock for pigment extraction. Ultrasound-assisted extraction (UAE) provided 0.124 g bixin per g of seeds, while the complementary low-pressure solvent extraction (LPSE) of fine seed fractions contributed an additional 0.100 g g^–1^, underscoring the potential of combining innovative and conventional solvent-based techniques to achieve high pigment recovery.

Beyond oil and pigments, the depigmented seed biomass was further valorized into starch, with an average yield of 0.66 g g^–1^ (dry basis). Previous studies have demonstrated that annatto starch displays distinct physicochemical and technological properties, such as high swelling power and thermal stability, which open avenues for its use in food formulations and biodegradable materials.^5,6,19^ By integrating starch recovery into the process design, the scheme avoids discarding the pigment-depleted residue, instead transforming it into a value-added carbohydrate fraction.

Finally, the oil extract was structured into nutraceutical powders through ultrasound-assisted emulsification followed by freeze- or spray-drying. The obtained microparticles reached yields of 0.25 g g^–1^ (freeze-dried) and 0.18 g g^–1^ (spray-dried), resulting in stable powders with potential for dietary supplement applications. This final step illustrates the functional diversification achievable by coupling encapsulation technologies with extraction processes, thereby extending market opportunities from food and cosmetics to the nutraceutical sector.

These integrated results demonstrate the multiproduct potential of annatto seeds when processed under an integrated biorefinery concept. While the yields of individual fractions may appear modest when analyzed in isolation, their cumulative recovery in a sequential framework transforms annatto from a traditional dye source into a versatile feedstock for sustainable ingredient production. This holistic valorization not only enhances the economic outlook of the process but also positions annatto seeds as a model biomass for future biorefineries aimed at delivering natural pigments, functional carbohydrates, and nutraceutical bioactives.

### 3.2 Capital Investment and Equipment Requirements

The economic feasibility of any emerging biorefinery is strongly determined by its capital expenditures (CAPEX), which encompass direct equipment costs, installation, and auxiliary infrastructure. In the proposed annatto seed biorefinery, capital investment was estimated by scaling laboratory-scale equipment costs (Table 3) using standard power-law correlations and applying multipliers for installation, piping, and indirect costs (Table 2). This methodology allowed for a realistic projection of industrial-scale requirements while accounting for the technological complexity of each unit operation.

Among the equipment items, the high-pressure extraction vessel and CO_2_ pump for supercritical CO_2_ extraction, together with drying units (freeze-dryer and spray dryer), emerged as the dominant contributors to total capital cost. These systems represent more than half of the total plant direct cost, reflecting both the engineering sophistication and the robust construction materials required for high-pressure and high-temperature operation. While capital-intensive, these units are indispensable for ensuring food-grade, solvent-free extracts, which carry significant commercial premiums in the natural colorant and nutraceutical markets. Moreover, supercritical CO_2_ systems are modular and flexible, enabling their use with a wide range of botanical feedstocks beyond annatto seeds, thereby increasing the long-term value of the investment through multipurpose operation.

Freeze-drying, though associated with high equipment cost (USD 3730 base cost per unit at laboratory scale), was included in the design due to its ability to generate nutraceutical powders with superior retention of sensitive bioactives. Spray-drying, a comparatively less capital-demanding alternative (USD 4800 base cost for higher throughput units), complements the process by enabling production of encapsulates at larger scales with lower operational costs. The inclusion of both technologies highlights the strategy of combining premium, small-volume nutraceutical products (freeze-dried powders) with more scalable, cost-effective outputs (spray-dried powders), thereby addressing diverse market demands.

Auxiliary units, such as centrifuges, mills, rotary dryers, and filtration systems, contributed less to the overall investment but remain critical for ensuring continuous and reliable operation. These units are relatively inexpensive when compared to high-pressure and drying equipment, yet they enable the integrated valorization chain by preparing defatted seeds, facilitating pigment and starch recovery, and ensuring proper material handling. Importantly, their modular nature allows straightforward adaptation to different particle sizes, feedstock properties, and downstream applications.

The resulting total plant cost structure underscores a key feature of clean-label and green-processing technologies: the trade-off between higher upfront investments and the ability to access high-value product markets with reduced environmental and regulatory burdens. Although such investments may present entry barriers for small- and medium-scale enterprises, the long-term competitiveness of biorefineries increasingly depends on technologies that deliver solvent-free extracts, energy-efficient operations, and compliance with sustainability standards. Thus, while CAPEX represents a significant portion of the financial commitment in the proposed scheme, it also ensures technological robustness, product diversification, and regulatory alignment, positioning the annatto seed biorefinery as a competitive alternative to conventional solvent-intensive facilities.

### 3.3 Operating Costs and Resource Distribution

Beyond capital expenditures, the long-term competitiveness of a biorefinery depends primarily on its operating costs (OPEX), which determine annual profitability and resilience to market fluctuations. In the proposed annatto seed biorefinery, operating costs were broken down into raw materials, labor, and utilities (Table 4), with their relative contributions illustrated in Figure 3.

Material costs emerged as the dominant category, accounting for the largest share of total expenditures, as it occurred in other works.^20^ The annatto seeds themselves, priced at USD 2.78 kg^–1^, represented the single most significant cost driver, followed by solvents such as ethanol and CO_2_. This dependency highlights a common challenge in biomass-based industries: feedstock volatility. Seasonal availability, fluctuations in agricultural yields, and competing uses of annatto seeds for pigment extraction can directly influence cost structures, making the stability of raw material supply a decisive factor for economic success.

In addition to seeds and solvents, functional excipients used for oil encapsulation, such as whey protein isolate (USD 9.60 kg^–1^), gum Arabic (USD 9.20 kg^–1^), and inulin (USD 13.50 kg^–1^), introduced further costs. Although their unit prices are relatively high compared to the bulk raw material, these inputs enable the production of differentiated nutraceutical powders, which command higher selling prices and open access to premium markets. This trade-off exemplifies the economic logic of product functionalization: investing in costly inputs to create value-added formulations with greater commercial appeal.

Labor and utility costs, in contrast, played a comparatively minor role in the overall OPEX. Electricity (USD 0.25 (kW h)^−1^) and steam (USD 12.00 MT^–1^) together accounted for a modest fraction of total costs, underscoring the relative energy efficiency of the proposed process chain. Labor costs, although relevant, were proportionally less impactful than raw materials, especially under the integrated design that allows process steps to be executed in parallel and with reduced manpower requirements. This observation reinforces that, in biorefineries based on agricultural feedstocks, the financial risk is less associated with workforce or energy expenses and more strongly tied to raw material markets.

The cost distribution analysis provides critical insights for process optimization and risk management. First, strategies to stabilize annatto seed supply, such as contractual agreements with producers or valorization of alternative biomass fractions, are essential for mitigating feedstock-related risks. Second, solvent recycling and process intensification could further reduce material consumption, particularly in pigment extraction steps. Third, the use of multifunctional encapsulation agents or replacement with lower-cost alternatives may reduce reliance on high-priced additives, without compromising product quality.

Overall, the operating cost profile of the integrated annatto seed biorefinery highlights a dual dynamic: while seed costs and excipients drive the largest expenditures, these same inputs are responsible for generating premium-value products that strengthen profitability. This balance between cost intensity and product differentiation lies at the core of sustainable biorefining, ensuring not only economic viability but also alignment with consumer demand for natural, clean-label, and multifunctional ingredients

### 3.4 Profitability Indicators

The profitability of the integrated annatto seed biorefinery was assessed using standard financial indicators, including GM, ROI, net present value (NPV), and internal rate of return (IRR). Under the baseline scenario, the process achieved a GM of 21.8%, ROI of 27.9%, and an IRR of 19.1%, with an estimated NPV of USD 5.8 million at a 7% discount rate (Table 5). These values place the proposed scheme well within the range considered attractive for agro-industrial ventures, particularly in comparison to traditional commodity-based operations that typically struggle to exceed double-digit ROI.

**Table 5:** GM, ROI, IRR, NPV, and GP for the Scenarios Evaluated for Processing One Ton of Annatto Seeds Per Batch^a^

Scenario	GM (%)	ROI (%)	IRR (%)	NPV at 7% interest (10^3^ USD)	GP (10^3^ USD year^‑1^)
Standard	21.77	27.90	19.14	5765	2039
Labor(+5%)	21.19	27.35	18.52	5489	1985
Labor(+20%)	19.45	25.72	16.80	4661	1822
Labor(+35%)	17.71	24.10	15.23	3833	1659
Labor(−5%)	22.35	28.45	19.77	6041	2094
Labor(−20%)	24.09	30.09	21.48	6869	2257
Labor(−35%)	25.83	31.75	23.2	7697	2420
Utility(+5%)	21.47	27.62	18.98	5643	2011
Utility(+20%)	20.59	26.78	18.20	5281	1928
Utility(+35%)	19.70	25.95	17.58	4919	1845
Utility(−5%)	22.07	28.18	19.45	5884	2067
Utility(−20%)	22.95	29.01	20.08	6244	2150
Utility(−35%)	23.83	29.84	20.70	6603	2232
Material(+5%)	18.47	24.80	16.64	4419	1730
Material(+20%)	11.83	18.65	11.02	1710	1108
Material(+35%)	4.19	11.73	3.67	–1274	393
Material(−5%)	23.98	29.99	20.86	6664	2246
Material(−20%)	31.72	37.4	26.33	9819	2971
Material(−35%)	38.26	43.79	30.86	12485	3584
Selling(+5%)	25.50	32.42	22.73	7782	2508
Selling(+20%)	34.81	45.98	32.58	13835	3913
Selling(+35%)	42.05	59.54	41.33	19888	5318
Selling(−5%)	17.66	23.38	15.23	3747	1571
Selling(−20%)	2.22	9.82	0.86	–2201	166
Selling(−35%)	–20.35	–11.71	NR	–11542	–1239

a NR: No return.

Values of GP were estimated at approximately USD 2.0 million per year, positioning the process alongside medium-scale natural product facilities. Importantly, these outcomes were achieved while adhering to green processing principles, indicating that environmental responsibility does not compromise financial performance. Instead, the integration of supercritical CO_2_ extraction, ultrasound/solvent-assisted pigment recovery, and aqueous starch isolation generated a portfolio of products that collectively enhanced economic robustness.

The breakdown of operating costs and revenues (Figure 4A) underscores the relative stability of the integrated process. Even when labor and utility costs were varied, net profits remained resilient (Figure 4B), confirming that these factors exert comparatively minor effects on overall profitability. This finding aligns with the analysis of operating costs (Section 3), where raw materials and selling prices emerged as the true determinants of economic success, and shown elsewhere for other purposes of biorefineries.^21^

When benchmarked against reported values for other biorefineries, the annatto seed system shows a competitive advantage. For instance, lignocellulosic ethanol plants often report ROI values below 15% due to high feedstock and enzyme costs, while essential oil facilities typically achieve ROI between 20 and 25%. By surpassing these benchmarks, the integrated annatto process demonstrates that multiproduct valorization not only diversifies revenue streams but also significantly improves financial attractiveness.

Another critical insight is that profitability stems from the complementarity of product streams rather than reliance on a single high-value fraction. While annatto oil and bixin represent the most lucrative products, starch and nutraceutical encapsulates contribute to stabilizing revenues, especially under market fluctuations. This diversification effect confirms that integrated designs mitigate the risks associated with commodity price volatility, reinforcing the resilience of the business case.

The integrated financial indicators provide a compelling argument for the viability of the proposed scheme. The combination of robust GM, ROI, and IRR values with a positive NPV highlights that the integrated annatto seed biorefinery is not only technically feasible but also financially competitive within the natural product industry. This profitability, achieved in alignment with sustainability goals, represents a crucial step toward the industrial translation of clean-label and zero-waste biorefining concepts.

### 3.5 Sensitivity Analysis

A sensitivity analysis was conducted by varying operating costs (materials, labor, utilities) and product selling prices by ± 5, ± 20, and ± 35% to further assess the robustness of the proposed biorefinery. The results (Table 5 and Figure 4B) highlight that profitability is strongly governed by raw material costs and product prices, while fluctuations in labor and utilities have comparatively minor impacts.

Variations in labor costs produced only modest changes in financial indicators. A 35% increase in labor reduced ROI from 27.9% to 24.1%, with IRR still above 15%, confirming that the system remains economically viable even under pessimistic labor market scenarios. Similarly, utility cost increases led to ROI values above 25% and GM consistently above 19%, underscoring the limited influence of energy and water prices on overall profitability. This resilience reflects the relatively low energy intensity of the proposed integrated chain, where solvent-based and mechanical steps dominate resource consumption.

By contrast, the effect of feedstock costs was far more pronounced. A 20% increase in annatto seed price reduced NPV by almost 70% and ROI to 18.7%, while a 35% increase rendered the project nearly unprofitable, with NPV turning negative (−USD 1.3 million). Conversely, a 20% reduction in seed cost nearly doubled profitability, elevating ROI above 37% and NPV close to USD 10 million. This asymmetry highlights the centrality of feedstock economics in biomass-based industries, where raw material expenses can represent over half of total production costs. It also reinforces the need for strategic procurement policies, long-term contracts with producers, or valorization of alternative biomass fractions to reduce dependency on seed price fluctuations.

Perhaps the most decisive factor influencing profitability was product selling prices. A 20% increase across product streams nearly doubled the NPV (to USD 13.8 million) and boosted ROI to 46%, while a 35% increase pushed ROI beyond 59%. In contrast, a 20% decrease reduced ROI to nearly zero, and a 35% decrease rendered the project economically unfeasible, with a strongly negative NPV (−USD 11.5 million). This sharp asymmetry illustrates the double-edged nature of high-value natural products: while clean-label pigments, nutraceuticals, and functional carbohydrates benefit from market premiums, volatility in consumer demand or oversupply could rapidly erode profitability.

The sensitivity analysis clearly identifies selling prices and raw material costs as the dominant profitability drivers, while labor and utility costs remain secondary considerations. From a strategic perspective, this finding highlights the need for risk management measures, including product diversification, forward contracts, and positioning in premium markets less susceptible to commoditization. Importantly, the integrated process design distributes risks across multiple products, oil, bixin, starch, and encapsulates, providing a built-in buffer against market volatility.

In summary, the sensitivity analysis reveals both the opportunities and vulnerabilities of the proposed biorefinery. Under favorable conditions of stable feedstock supply and strong product prices, the system is highly profitable and resilient. However, under adverse scenarios, particularly involving sharp decreases in selling prices, profitability can be compromised. This duality underscores the importance of aligning technological innovation with robust market strategies to ensure the long-term success of annatto seed valorization.

### 3.6 Payback Period and Risk Mitigation

The payback period is a critical metric for investors, as it directly reflects the time required to recover the initial capital investment.^22^ Under standard operating conditions, the proposed annatto seed biorefinery demonstrated a payback period of approximately 3–4 years (Figure 5). This time frame is considered highly attractive in the food and nutraceutical sectors, where payback horizons of 5–7 years are typically expected for greenfield facilities. The relatively short recovery period strengthens the case for industrial adoption, particularly in regions where capital availability and financial risk perception are major determinants of project implementation.

**Figure 5:**
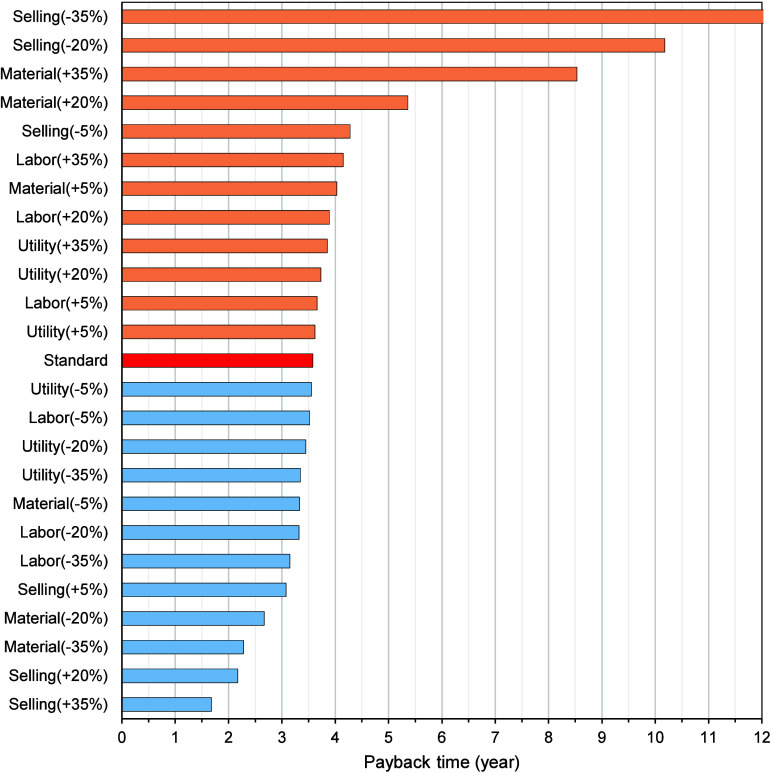
Payback time for the scenarios evaluated for processing one ton of annatto seeds per batch.

Favorable market scenarios further enhanced this outlook. A 20% increase in product selling prices reduced the payback period to less than 2 years, while the combined effects of lower raw material costs and higher product prices yielded recovery times close to 1.5 years. Such rapid capital recovery not only reflects strong economic resilience but also provides flexibility for reinvestment in process optimization, product development, or expansion into other biomass feedstocks.

In contrast, unfavorable conditions, especially decreases in product selling prices, dramatically extended the payback period or, in extreme cases (−35% scenario), eliminated the possibility of recovery. This result mirrors the findings of the sensitivity analysis (Section 5), reinforcing that product price volatility is the single most decisive factor shaping financial feasibility. These scenarios highlight the fragility of profit margins when market demand or competitive pressures drive down selling prices, a common risk in the natural colorant and nutraceutical industries.

From a risk mitigation perspective, the integrated design of the process provides an inherent advantage. By diversifying outputs across multiple product categories, natural pigments (bixin), bioactive-rich oil, functional carbohydrates (starch), and nutraceutical encapsulates, the scheme reduces dependency on a single revenue stream. This “portfolio effect” provides a financial buffer: if demand contracts for one product, revenues from other streams can sustain overall cash flow. In addition, valorization of residual fibers, although not included in the current economic assessment, represents a further opportunity for strengthening resilience by generating additional dietary or biomaterial products.

Overall, the payback analysis highlights the dual nature of the annatto seed biorefinery: while highly profitable and fast in capital recovery under favorable conditions, it remains vulnerable to adverse market shifts. The success of its implementation will therefore rely not only on efficient process integration but also on robust business strategies, such as long-term supply agreements, vertical integration with food or cosmetic companies, and continued diversification of the product portfolio.

### 3.7 Integration as a Value Creation Strategy

A defining strength of the proposed biorefinery lies in its integrated process design, where multiple unit operations are aligned both sequentially and in parallel to maximize biomass utilization and minimize waste. The Gantt chart (Figure 6) illustrates the temporal organization of extraction, drying, and encapsulation steps, showing how the valorization of different seed fractions can proceed synergistically within a coordinated schedule. Such integration not only enhances operational efficiency but also reduces idle times between processing stages, ultimately improving throughput and resource utilization.

**Figure 6:**
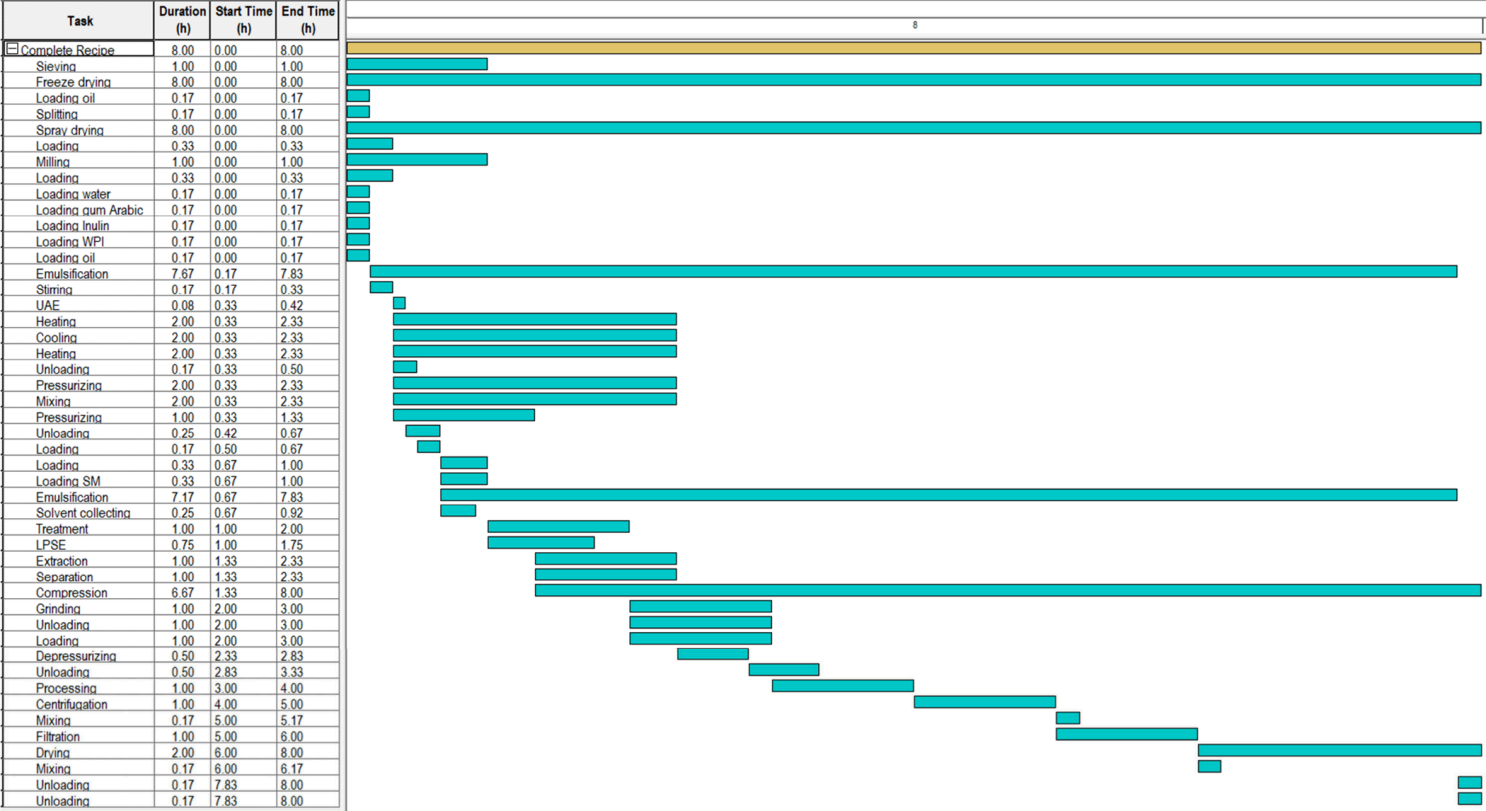
Gantt chart of the integrated processes for annatto seeds processing.

From an economic perspective, integration redistributes fixed and operating costs across several product streams.^23^ Instead of a single-product operation vulnerable to price volatility, the annatto seed biorefinery generates a diversified portfolio: bixin pigments, bioactive-rich oil, functional starch, and nutraceutical powders. This portfolio effect stabilizes revenues, ensuring that fluctuations in one market segment (e.g., colorants) can be offset by stability or growth in others (e.g., nutraceuticals or carbohydrates). The diversification advantage was evident in the sensitivity and payback analyses, where multiproduct valorization consistently mitigated financial risks under adverse scenarios.

Integration also reinforces the sustainability profile of the process. The sequential exploitation of the biomass ensures that every fraction of the seed contributes to value generation. Oil is extracted first, followed by pigments from the defatted material, starch from the depigmented residue, and encapsulates from the oil fraction. Only a small fiber-rich fraction remains unvalorized, which itself holds promise for dietary fiber or biomaterial applications. This approach exemplifies a zero-waste strategy, transforming annatto from a conventional pigment source into a multipurpose raw material for clean-label and circular economy applications.

Beyond economic and environmental benefits, integration supports industrial scalability by promoting modularity. Each unit operation, supercritical CO_2_ extraction, ultrasound-assisted pigment recovery, starch isolation, and encapsulation, can be individually optimized or scaled according to market demand, while still fitting into the broader valorization chain. This flexibility is particularly relevant for emerging biobased industries,^24^ where dynamic adaptation to evolving consumer trends (e.g., clean-label food additives, functional beverages, nutraceutical supplements) is crucial for competitiveness.

In summary, the integrated process design of the annatto seed biorefinery is not merely a technological configuration but a value creation strategy. By combining product diversification, cost redistribution, waste minimization, and modular scalability, integration elevates the biorefinery from a collection of unit operations to a coherent and resilient business model aligned with sustainability principles.

### 3.8 Sustainability Assessment by Path2Green

While profitability is essential for industrial adoption, the long-term success of a biorefinery also depends on its environmental and social responsibility. To capture these dimensions, the extraction stages of the annatto seed biorefinery were evaluated using Path2Green, a recently proposed metric based on the 12 principles of green extraction.^18^ This framework allows benchmarking of different technologies by considering solvent safety, energy demand, scalability, waste management, and application potential, ultimately translating complex environmental data into an accessible pictogram-based score (Figure 7).

**Figure 7:**
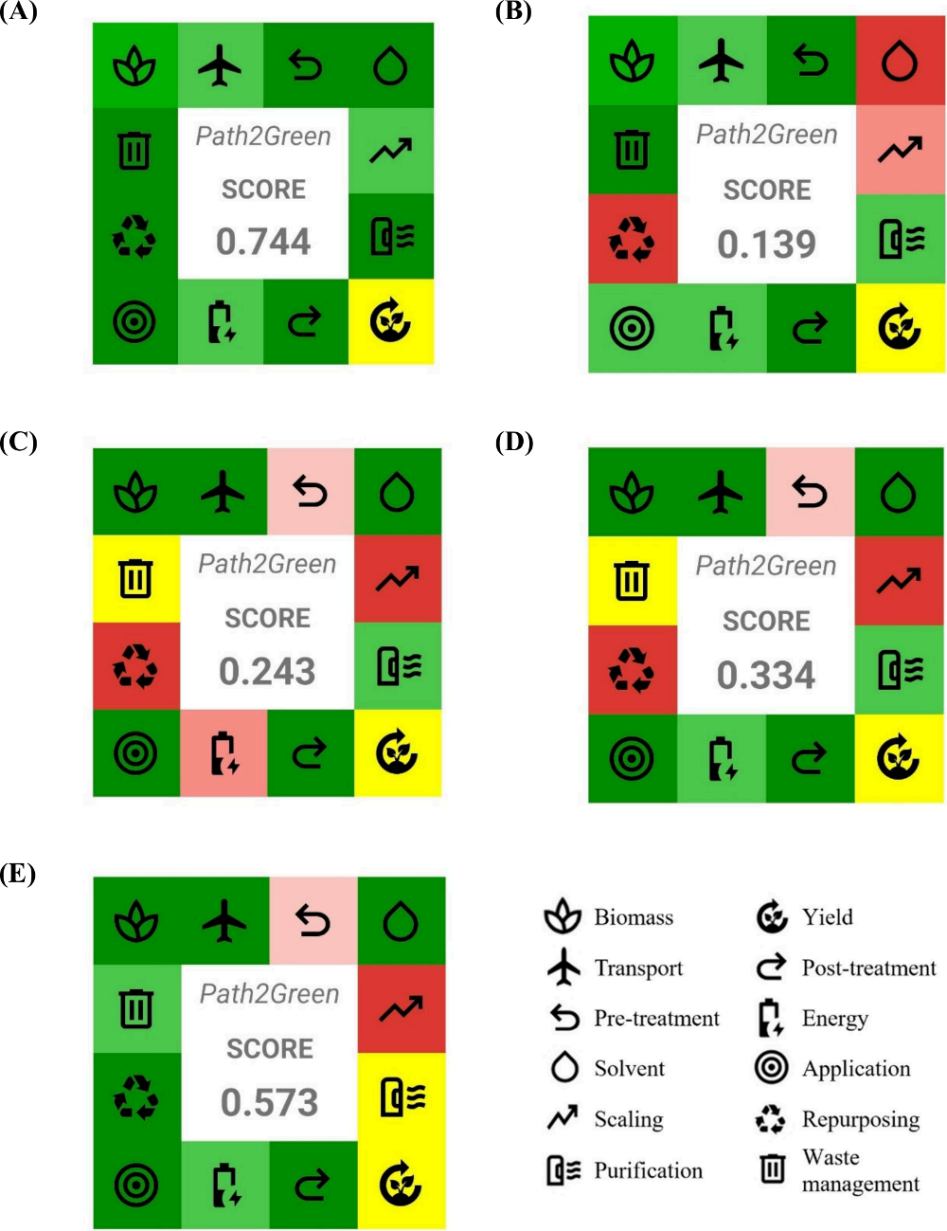
Pictograms representing the Path2Green sustainability scores for the extraction processes evaluated in the integrated annatto seed biorefinery. (A) Supercritical CO_2_ extraction of annatto seed oil, (B) Soxhlet extraction of annatto seed oil, (C) ultrasound-assisted extraction of bixin, (D) low-pressure solvent extraction of bixin, and (E) aqueous extraction of starch. The pictograms summarize the performance of each process across the 12 green extraction principles.

The comparison between supercritical CO_2_ extraction and conventional Soxhlet highlights how solvent choice decisively shapes sustainability outcomes (Figure 7A and B). CO_2_, classified as a safe, recyclable, and residue-free solvent, enabled the production of oil directly suitable for food, nutraceutical, and cosmetic markets without additional purification. This advantage is reflected in the higher Path2Green score for SFE, which also benefits from its semicontinuous configuration and industrial scalability. In contrast, Soxhlet extraction with hexane was penalized due to the use of a hazardous solvent, the need for downstream purification, and limited scalability beyond laboratory settings. Although both methods provided exhaustive yields (generally >90%), only SFE aligned fully with green extraction principles, reinforcing its role as the benchmark technology for solvent-free bioactive recovery.

The Path2Green analysis also compared the UAE and LPSE for pigment recovery (Figure 7C and D). Both routes benefited from the use of ethanol, a recommended solvent, and the valorization of defatted seeds, which minimized waste and eliminated transport requirements. However, the sustainability profiles diverged in terms of energy demand. UAE, despite accelerating mass transfer and improving kinetics, was penalized for its higher power consumption, even under scenarios where hydropower could mitigate the carbon footprint. LPSE, in contrast, was classified as less energy-intensive, resulting in a higher score for the energy principle. This trade-off highlights that while the UAE may deliver superior extraction efficiency, its environmental impact must be weighed against simpler, lower-energy alternatives when scaling up.^25^

The third valorization step, aqueous extraction of starch from depigmented seeds, scored positively across most principles, particularly in solvent choice, scalability, and industrial applicability (Figure 7E). The use of water as the main solvent aligns perfectly with green extraction guidelines, and the resulting starch holds broad applications in food and packaging.^26^ However, two bottlenecks emerged: (*i*) the process generated a measurable waste fraction (∼30%) in the form of residual fibers, and (*ii*) ethanol washing was required during purification, lowering the score compared to a fully solvent-free system. Although these constraints reduced the overall sustainability index, they also represent opportunities for future innovation, particularly through the valorization of residual fibers as dietary ingredients or biomaterials.

Perhaps the most important insight of the Path2Green assessment is that integration enhances sustainability at the system level.^27^ By sequentially valorizing oil, pigments, and starch, the biorefinery achieved near-complete biomass utilization, minimizing waste and approaching a closed-loop system. Each stage builds upon the residues of the previous one, demonstrating how circularity can be embedded directly into process design. Moreover, the sustainability trade-offs identified at the unit operation level (e.g., higher energy demand of the UAE, partial waste generation in starch recovery) are mitigated when considered in the broader context of the integrated chain. This confirms that the annatto seed biorefinery not only delivers financial returns but also aligns with the principles of sustainable chemistry and engineering.

The Path2Green evaluation adds a crucial dimension to the techno-economic analysis: it demonstrates that profitability and sustainability are not mutually exclusive but can be synergistically achieved in well-designed biorefineries. SFE was confirmed as the most sustainable extraction option, LPSE provided a low-energy alternative to UAE for pigment recovery, and starch extraction illustrated the value of waste conversion while highlighting areas for improvement. Together, these findings provide a roadmap for optimizing the process according to both environmental and economic criteria. Importantly, they also position annatto seeds as a model biomass for demonstrating how traditional natural products can be transformed into multistream, circular value chains aligned with global sustainability goals.

Although Path2Green provides a structured and transparent comparison of extraction technologies based on green chemistry and engineering principles, its scope remains limited to process-level evaluation. Therefore, the sustainability advantages observed for certain technologies, such as supercritical CO_2_ extraction, should be interpreted within the defined system boundaries. A comprehensive cradle-to-gate Life Cycle Assessment would be required to fully quantify environmental trade-offs, including greenhouse gas emissions, cumulative energy demand, and water footprint, and to support decision-making at industrial scale.

## 4 Conclusion

This study demonstrated the feasibility of an integrated biorefinery for annatto (*Bixa orellana* L.) seeds, designed to sequentially generate high-value products including oil, bixin pigments, starch, and nutraceutical powders. By coupling supercritical CO_2_ extraction, ultrasound- and solvent-assisted pigment recovery, aqueous starch isolation, and oil encapsulation, the process maximized biomass utilization and minimized waste, fully aligning with zero-waste and circular economy principles.

Techno-economic analysis revealed that the integrated scheme is financially attractive, with a gross margin of 21.8%, ROI of 27.9%, NPV of USD 5.8 million, and a payback period of 3–4 years under baseline conditions. Sensitivity analysis identified raw material costs and product prices as the dominant profitability drivers, while labor and utilities exerted minor influence. Importantly, the multiproduct design mitigated risks associated with market volatility, reinforcing the economic resilience of the system.

Sustainability evaluation using the Path2Green metric confirmed that environmental responsibility can be achieved alongside profitability. Supercritical CO_2_ extraction outperformed Soxhlet due to solvent safety and scalability, LPSE provided a low-energy alternative to ultrasound-assisted pigment recovery, and aqueous starch extraction highlighted both the potential of residue valorization and opportunities for future optimization. These results show that methodological choices decisively shape the sustainability profile of each unit operation, but integration at the system level ensures alignment with green extraction principles.

Overall, the integrated annatto seed biorefinery emerges as a model platform for producing natural pigments, functional carbohydrates, and nutraceutical bioactives in a sustainable and economically viable manner. Beyond demonstrating technical feasibility, this work highlights the strategic role of combining techno-economic and sustainability metrics to guide the development of next-generation biorefineries.

It is important to acknowledge that the sustainability assessment presented in this study is limited to process-level indicators and does not constitute a full cradle-to-gate life cycle assessment. While the Path2Green metric provides a structured, principle-based evaluation of solvent selection, energy intensity, scalability, and waste generation within the extraction stages, it does not capture upstream and downstream burdens such as biomass cultivation, CO_2_ production and recycling, embodied energy in equipment, or end-of-life impacts. Future studies should therefore integrate a comprehensive life cycle assessment framework to quantify greenhouse gas emissions, cumulative energy demand, water use, and potential trade-offs across the integrated biorefinery, enabling a more holistic comparison with conventional technologies and supporting decision-making at industrial and policy levels.

## References

[1] Fulignati S., Raspolli Galletti A.
M., Barsotti F., Menicagli V., Balestri E., Lardicci C., Mattonai M., Nardella F., Antonetti C. (2025). Sustainable Exploitation of Posidonia
oceanica Balls through an Integrated Biorefinery Approach. ACS Sustainable Chem. Eng..

[2] Coelho dos Santos D., Silva Barboza A. d., Ribeiro J. S., Rodrigues Junior S. A., Campos Â. D., Lund R. G. (2022). Bixa orellana L. (Achiote, Annatto) as an antimicrobial
agent: A scoping review of its efficiency and technological prospecting. Journal of Ethnopharmacology.

[3] Strieder M. M., Vardanega R., Moraes M. N., Silva E. K., Meireles M. A. A. (2024). One-step
ultrasound-assisted recovery of yellow-orange-red natural coloring
from defatted annatto seeds: A cleaner processing alternative. Ultrasonics Sonochemistry.

[4] Shahid-ul-Islam, Rather L. J., Mohammad F. (2016). Phytochemistry,
biological activities and potential of annatto in natural colorant
production for industrial applications – A review. Journal of Advanced Research.

[5] Zabot G. L., Silva E. K., Emerick L. B., Felisberto M. H. F., Clerici M. T. P. S., Meireles M. A. A. (2019). Physicochemical,
morphological, thermal and pasting properties of a novel native starch
obtained from annatto seeds. Food Hydrocolloids.

[6] Silva E. K., Anthero A. G. d. S., Emerick L. B., Zabot G. L., Hubinger M. D., Meireles M. A. A. (2022). Low-frequency
ultrasound-assisted esterification of *Bixa orellana* L. seed starch with octenyl succinic anhydride. Int. J. Biol. Macromol..

[7] Albuquerque C. L. C., Meireles M. A. A. (2012). Defatting of
annatto seeds using
supercritical carbon dioxide as a pretreatment for the production
of bixin: Experimental, modeling and economic evaluation of the process. J. Supercrit. Fluids.

[8] Moraes M. N., Zabot G. L., Meireles M. A. A. (2015). Extraction of
tocotrienols from annatto
seeds by a pseudo continuously operated SFE process integrated with
low-pressure solvent extraction for bixin production. J. Supercrit. Fluids.

[9] Alcázar-Alay S. C., Osorio-Tobón J. F., Forster-Carneiro T., Meireles M. A. A. (2017). Obtaining bixin from semi-defatted
annatto seeds by
a mechanical method and solvent extraction: Process integration and
economic evaluation. Food Research International.

[10] Silva E. K., Azevedo V. M., Cunha R. L., Hubinger M. D., Meireles M. A. A. (2016). Ultrasound-assisted
encapsulation of annatto seed oil: Whey protein isolate versus modified
starch. Food Hydrocolloids.

[11] Silva E. K., Meireles M. A. A. (2015). Influence of
the degree of inulin polymerization on
the ultrasound-assisted encapsulation of annatto seed oil. Carbohydr. Polym..

[12] Zabot G. L., Moraes M. N., Meireles M. A. A. (2018). Process integration
for producing
tocotrienols-rich oil and bixin-rich extract from annatto seeds: A
techno-economic approach. Food and Bioproducts
Processing.

[13] Silva E. K., Zabot G. L., Meireles M. A. A. (2015). Ultrasound-assisted encapsulation
of annatto seed oil: Retention and release of a bioactive compound
with functional activities. Food Research International.

[14] Heinzle, E. ; Biwer, A. P. ; Cooney, C. L. Development of Sustainable Bioprocesses: Modeling and Assessment; John Wiley & Sons, 2006.

[15] Cheng M.-H., Rosentrater K. A. (2017). Economic feasibility analysis of soybean oil production
by hexane extraction. Industrial Crops &
Products.

[16] Turton, R. ; Bailie, R. C. ; Whiting, W. B. ; Shaeiwitz, J. A. ; Bhattacharyya, D. Analysis, Synthesis and Design of Chemical Processes; Prentice Hall, 2012.

[17] Ulrich, G. A Guide to Chemical Engineering Process Design and Economics; John Wiley & Sons Inc, 1984.

[18] de
Souza Mesquita L. M., Contieri L. S., e Silva F. A., Bagini R. H., Bragagnolo F. S., Strieder M. M., Sosa F. H. B., Schaeffer N., Freire M. G., Ventura S. P. M. (2024). Path2Green: introducing
12 green extraction principles and a novel metric for assessing sustainability
in biomass valorization. Green Chem..

[19] Cortés-Viguri V., Hernández-Rodríguez L., Lobato-Calleros C., Cuevas-Bernardino J.
C., Hernández-Rodríguez B. E., Alvarez-Ramirez J., Vernon-Carter E. J. (2022). Annatto (Bixa orellana L.), a potential
novel starch source: antioxidant, microstructural, functional, and
digestibility properties. Journal of Food Measurement
and Characterization.

[20] Farias C. A. A., Camponogara J. A., dos Reis A. R., Schlesner S. K., Zabot G. L., de Moraes D. P., Bettio L., Schmiele M., Barin J. S., Ballus C. A. (2025). Combined use of microwaves in the simultaneous production of dehydrated
blueberries and aqueous extract. Food Chem..

[21] Pérez-Almada D., Galán-Martín Á., Contreras M. d. M., Romero-García J. M., Castro E. (2025). Uncovering the Techno-Economic and Environmental Implications
of a Multiproduct Biorefinery from Exhausted Olive Pomace. ACS Sustainable Chem. Eng..

[22] Maity S. K., Agrawal D., Gadkari S., Vanapalli K. R., Yong Y.-C., Zhu D., Chen C., Kumar V. (2025). Technoeconomics
of Sugar Cane Bagasse Valorization to Lactic Acid Using Pinch Technology:
Distillation vs Reactive Distillation. ACS Sustainable
Chem. Eng..

[23] Gilcher E. B., Lane M. K. M., Pontious R. S., Apatoff M. B. L., Ahrens-Víquez M. M., Zimmerman J. B. (2025). Sequential
Extraction and Purification of Triglycerides
and Carotenoids with Supercritical Carbon Dioxide for Valorization
of the Integrated Algal Biorefinery. ACS Sustainable
Chem. Eng..

[24] Zhou Y., Jia Y., Chang T., Zhao G., Huang Y., Yang W. (2025). Synthesis
of Cross-Linked Vegetable Oil Nanoparticles. ACS Sustainable Chem. Eng..

[25] Martins
Strieder M., Silva E. K. (2025). Eco-Friendly Genipin-Derived Blue
Food Colorants via Exhaustive Extraction of Unripe Genipap and Ultrasound-Assisted
Whey Protein Cross-Linking. ACS Food Science
& Technology.

[26] Milanezzi G. C., Silva E. K. (2025). Role of particle
size and co-extraction dynamics in
the sequential recovery of phenolics, starch, and proteins from potato
peel by-products. Food Research International.

[27] Bragagnolo F. S., Strieder M. M., Sanches V. L., de Souza Mesquita L. M., Barroso T. L. C. T., Forster-Carneiro T., Rostagno M. A. (2025). Stingless Bee Honeys
As Natural and Edible Extraction Solvents: An Intensified Approach
to Cocoa Bean Shell Valorization. ACS Sustainable
Chem. Eng..

